# *CDC Grand Rounds:* New Frontiers in Workplace Health

**DOI:** 10.15585/mmwr.mm6741a5

**Published:** 2018-10-19

**Authors:** Leah S. Fischer, Jason E. Lang, Ron Z. Goetzel, Laura A. Linnan, Phoebe Gates Thorpe

**Affiliations:** ^1^Division of Preparedness and Emerging Infections, National Center for Emerging and Zoonotic Infectious Diseases, CDC; ^2^Division of Population Health, National Center for Chronic Disease Prevention and Health Promotion, CDC; ^3^Johns Hopkins University Bloomberg School of Public Health, Baltimore, Maryland; ^4^IBM Watson Health, Cambridge, Massachusetts; ^5^Gillings School of Global Public Health, University of North Carolina, Chapel Hill; ^6^Office of Science, Office of the Deputy Director for Public Health Science and Surveillance, CDC.

## Overview of Current U.S. Workplace Health Promotion Programs

Approximately 150 million Americans go to work each day, and where and how they work are closely linked to health and disease. Thus, workplace health promotion programs provide an opportunity to affect the health of the nation. Workplace health promotion programs traditionally rooted in occupational safety and health focus on preventing injury and illness resulting from the workplace environment. As gains have been made in reducing workplace hazards, and the prevalence of disease has shifted toward chronic diseases, employers have encountered rising health care costs. In the United States, chronic diseases are responsible for approximately seven in 10 deaths and account for 86% of health care costs (*1*,*2*). Approximately 20% of employer health care spending is associated with 10 modifiable health risks in the U.S. workforce: depression, high blood glucose, high blood pressure, obesity, tobacco use, physical inactivity, high stress, high cholesterol, poor nutrition and eating habits, and high alcohol consumption (*3*). Many employers have sought to establish workplace health promotion programs to improve employee health and lower health care costs; results of these efforts have been mixed. For example, some employers, especially smaller firms with limited resources, report barriers to implementing workplace health promotion programs, including lack of knowledge of program design, difficulty identifying credible information, and lack of awareness of program benefits (*4*,*5*). Evaluation and research continue to increase knowledge about workplace health promotion program design and identify ways to overcome the challenges of establishing effective programs. State health departments can provide assistance to employers and employees. In 2017, the CDC Workplace Health Resource Center was launched as a source for reliable evidence and best practices to improve worker health and productivity, address research gaps, and potentially reduce health care costs.

Workplace health promotion programs are popular with both employers and employees, although programs offered by employers vary considerably. *Healthy People 2010* established five elements for a comprehensive workplace health promotion program, including 1) health education; 2) supportive social and physical environments; 3) integration of the worksite program into the organization’s culture; 4) links between health promotion and related programs like employee assistance; and 5) screenings with follow-up (*6*). A 2017 study based on two independent, nationally representative surveys of U.S. employers and employees (*7*) found that 81% of 705 surveyed employers offered some type of workplace health promotion program (Figure). The most frequently offered program elements were screenings with follow-up (70.4%), health education (64.3%), a supportive environment for health improvement (63.7%), and links to other employee services (50.4%). Using these same five elements, the 2015 Harris Poll Nielson survey found that a minority of employers (13.3%) offered comprehensive workplace health promotion programs (*7*).

**FIGURE:**
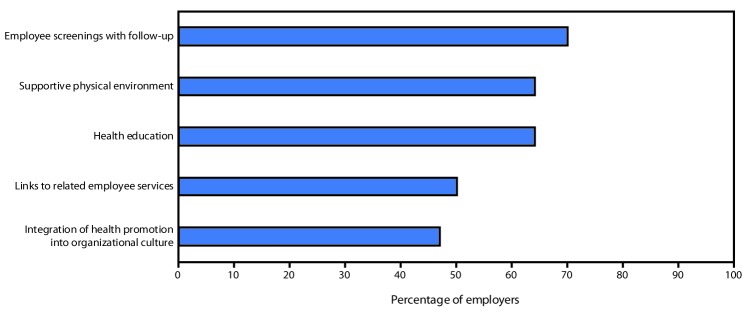
Percentage of employers offering the five elements included in workplace health promotion programs, by element — United States, 2017* * Figure adapted with permission from McCleary K, Goetzel RZ, Roemer EC, et al. Employer and employee opinions about workplace health promotion (wellness) programs: results of the 2015 Harris Poll Nielsen Survey. J Occup Environ Med 2017;59:256–63.

The existence of a workplace health promotion program, however, guarantees neither its use nor any resulting health and economic benefits. Among 1,833 employees surveyed by the 2015 Harris Poll Neilson survey, fewer than half (45%) reported being offered some form of workplace health promotion program, and 55% of those who were offered such a program reported participating (*7*). This gap between what employers offer and what employees perceive or use might reflect the variability in what program elements employers offer, or more likely, improperly designed programs that are not based on best or promising practices, or are underresourced or poorly implemented or both. Workplace health promotion programs that do not follow best practices, including assessing needs, often have low employee participation (*7*,*8*). However, accumulating evidence in the workplace health promotion program literature suggests that when these programs are well executed they benefit both employees and employers (*5*,*9*,*10*). In the 2015 Harris Poll Neilson survey, approximately three quarters of employers with a workplace health promotion program in place reported positive impacts from their wellness programs, including improved workers’ health (83.6%); performance and productivity (83.3%); and reduced health care costs (73.6%) (*7*). Survey results did not shed light on what made particular programs successful. A meta-analysis found that for every $1.00 spent on wellness programs, $3.27 was returned in reduced medical costs and $2.73 in absenteeism reductions (*11*). Research also has found reduced medical costs and absenteeism as well as fewer claims for short-term disability and safety/workers’ compensation (*12*–*14*).

## Workplace Health Promotion Program Evidence and Best Practices

Although employers have implemented programs and health departments have assisted through direct services to employers, gaps in understanding of workplace health promotion program best practices and evidence remain. In 2008 and 2013, reports sponsored by Partnership for Prevention and the Bipartisan Policy Center synthesized the evidence base from the field, described the need for and benefits of workplace health promotion programs, and provided actionable policy recommendations (*4*,*5*). These recommendations included improving employer education about benefits of workplace health promotion programs; providing technical assistance on the design, implementation, and evaluation of programs; developing and improving tools and resources to support these programs; and creating a comprehensive health promotion resource center.

Through its external workplace health promotion program, managed out of the National Center for Chronic Disease Prevention and Health Promotion, CDC was involved in several of the recommended activities, such as providing technical assistance and developing or improving tools and resources. However, no centralized resource for workplace health promotion existed.

## New CDC Workplace Health Resource Center

To fill this gap, and based on Partnership for Prevention and Bipartisan Policy Center recommendations, the CDC Workplace Health Resource Center (https://nccd.cdc.gov/WHRC/) was launched in August 2017, with the aim of serving as a comprehensive website with reliable information, tools, and resources to help employers find credible, public domain, fact-based resources from organizations already in the workplace health marketplace. All resources on the website are vetted by a steering committee comprising subject matter experts from state health departments, public and private sectors, and academia.

Structurally, the highest level of content is organized according to the CDC Workplace Health Model (assessment, planning and governance, implementation, and evaluation). Website users can search for resources within each of the model components. One notable feature of the Workplace Health Resource Center is the CDC Worksite ScoreCard (https://www.cdc.gov/workplacehealthpromotion/initiatives/healthscorecard/index.html), a comprehensive tool that employers can use to assess which health promotion activities are currently in place within an organization, plan strategies and interventions that could be implemented as part of a workplace health promotion program, and evaluate and monitor progress in primary health topic and programmatic areas.

Other search options on the website’s navigation bar include Workplace Organizational Factors (benefit plan design, creating a culture of health, etc.); Individual and Family Wellness (tobacco-free policies, healthy vending, and access to fitness facilities, etc.); Prevention Resources (clinical preventive services and vaccinations, etc.); and Health Conditions (disease management programs and lifestyle counseling to address chronic diseases, etc.) (Box). Users also can search for specific types of resources, including case studies; how-to manuals; peer-reviewed articles; and online, interactive training. Small businesses (those with fewer than 200 employees) might have difficulties offering a workplace health promotion program: whereas 55% of small businesses offer health insurance coverage, fewer than half offer wellness programs that address major lifestyle risks such as tobacco use and overweight/obesity (*15*). The website places a special emphasis on unique challenges and opportunities for small businesses, but can be used by all employers to tailor workplace health promotion programs to their organizations’ needs.

BOX Organization of the Workplace Health Resource Center*

**Organizational or employer factors**

- Creating a culture of health
- Employee engagement
- Strategic communication
- Benefit plan design
- Legal and regulatory environment
- Wellness and health promotion technology

**Individual or employee factors**

- Physical activity and fitness
- Nutrition
- Mental and emotional health
- Financial health
- Work-life balance
- Social connectedness

* https://nccd.cdc.gov/WHRC/

## State Health Departments’ Support of Workplace Health Promotion Programs

Within state health departments, occupational safety and health and workplace health promotion departments support and assist employers in implementing workplace health promotion programs. A 2017 national survey of Workplace Health Promotion and Occupational Safety and Health within health departments found that surveillance and implementation support were the activities most commonly reported by occupational safety and health and workplace health promotion program respondents, respectively (L Linnan, University of North Carolina, unpublished data, 2018). Implementation support might include providing technical assistance, training programs, educational materials/tools, and quality assurance/improvement. Fifty-one percent of survey respondents reported that their health department was involved in direct service to workers; occupational safety and health and workplace health promotion program respondents were equally likely (61%) to report this activity. Importantly, occupational safety and health programs in 26 health departments receive funding from CDC’s National Institute for Occupational Safety and Health to conduct occupational safety and health surveillance. However, many state health departments also reported that capacity to support occupational safety and health and workplace health promotion program activities is limited because of low funding and staffing levels: 19% of occupational safety and health and 30% of workplace health promotion program respondents indicated they had no funding designated for these efforts.

## The Role of Public Health in a 21st Century Workplace for a 21st Century Workforce

Chronic disease prevention and health promotion represent major challenges for employers in the 21st century. In aggregate, workplace health promotion programs can affect population health outcomes while improving individual quality of life and productivity. Evidence-based and best practice literature exists for the design, implementation, and evaluation of workplace health promotion programs. Dissemination to employers and health department programs that support employers in promoting occupational safety and health and workplace health promotion can encourage maximum effectiveness of workplace health promotion programs. Small and mid-size employers, particularly those without experience in workplace health, could benefit from information that is credible and useful. Support from CDC, state health departments, and professional organizations can facilitate acceptance of science-based strategies for workplace health promotion program development, implementation, and evaluation. Together, public health and employers can implement employer-based workplace health promotion programs to address modifiable health risks, lower the prevalence of chronic conditions, and improve the health and well-being of workers.
